# DNA intercalation optimized by two-step molecular lock mechanism

**DOI:** 10.1038/srep37993

**Published:** 2016-12-05

**Authors:** Ali A. Almaqwashi, Johanna Andersson, Per Lincoln, Ioulia Rouzina, Fredrik Westerlund, Mark C. Williams

**Affiliations:** 1 Department of Physics, Northeastern University, Boston, MA, 02115, USA; 2 Physics Department, King Abdulaziz University, Rabigh, 21911, Saudi Arabia; 3 Department of Chemistry-BMC, Uppsala University, Uppsala, SE-75123, Sweden; 4 Department of Chemistry and Chemical Engineering, Chalmers University of Technology, Gothenburg, SE-41296, Sweden; 5 Department of Chemistry and Biochemistry, Ohio State University, Columbus, OH, 43210, USA; 6 Department of Biology and Biological Engineering, Chalmers University of Technology, Gothenburg, SE-41296, Sweden

## Abstract

The diverse properties of DNA intercalators, varying in affinity and kinetics over several orders of magnitude, provide a wide range of applications for DNA-ligand assemblies. Unconventional intercalation mechanisms may exhibit high affinity and slow kinetics, properties desired for potential therapeutics. We used single-molecule force spectroscopy to probe the free energy landscape for an unconventional intercalator that binds DNA through a novel two-step mechanism in which the intermediate and final states bind DNA through the same mono-intercalating moiety. During this process, DNA undergoes significant structural rearrangements, first lengthening before relaxing to a shorter DNA-ligand complex in the intermediate state to form a molecular lock. To reach the final bound state, the molecular length must increase again as the ligand threads between disrupted DNA base pairs. This unusual binding mechanism results in an unprecedented optimized combination of high DNA binding affinity and slow kinetics, suggesting a new paradigm for rational design of DNA intercalators.

DNA intercalation represents an invasive, yet reversible, mode of DNA-ligand binding. These essential features of DNA intercalation allow a wide range of precisely modulated therapeutic and biotechnological applications. Conventional intercalators, such as acridine^1^ and ethidium^2^,^3^,^4^, bind into the DNA lattice by direct insertion of planar aromatic moieties between the base pairs, for which the primary rate limiting step is breathing of the DNA double helix. In contrast, unconventional intercalators require further DNA deformation during association in order to accommodate bulky non-intercalating moieties, for example fitting cyclic polypeptide chains in DNA grooves, or breaking base pairs to thread a bulky moiety through DNA, before reaching the final intercalated state^5^,^6^,^7^. This strong DNA deformation is the primary rate-limiting step, giving unconventional intercalators much slower binding and dissociation from the final intercalated state, which is a desirable property for many DNA applications, including anti-cancer drugs^8^,^9^,^10^.

Figure 1A shows the equilibrium dissociation constant and the dissociation rate from the final intercalative state for all the DNA intercalators, including the intercalating system we report here, that have been studied by single-molecule force spectroscopy, a reliable method for quantitatively determining intercalation affinity and kinetics. It shows clusters of two different types of ligands. The fast (conventional) intercalators have dissociation time constants ranging from milliseconds to seconds, and the slow (unconventional) intercalators have dissociation time constants ranging from tens of seconds to tens of minutes. This plot illustrates the distinct nature of each type of intercalating system in terms of dissociation rates, governed by two different regimes of DNA structural fluctuations. Based on the available equilibrium and kinetic single molecule studies of DNA intercalators^5^,^7^,^11^,^12^,^13^,^14^, for cyanine dyes (cyan symbols), all but YOYO are conventional intercalators, while polypeptide intercalators (red), and threading intercalators (purple) are unconventional intercalators. We consider YOYO to be an unconventional intercalator because its relatively long linker must be accommodated before its second moiety is fully intercalated, resulting in overall slower intercalation relative to conventional intercalators^12^.

In this work we introduce a new mechanism of DNA intercalation, where the intercalating moiety is converted from a fast assembling conventional intercalative state to a slow assembling final intercalative state. This intercalative conversion is characterized for the rotationally flexible binuclear ruthenium complex [μ-bipb(phen)_4_Ru_2_]^4+^ (Pi) shown in Fig. 1B^15^,^16^. Considering the structure of Pi, note that it has the same bulky side groups in a similar right-handed Δ chirality as for the previously reported threading mono-intercalator P^5^ and threading bis-intercalator Pc^11^, but it has a different bridging moiety (Fig. 1B, shared bulky side group in black, different bridging moieties in green, blue, and orange for P, Pc and Pi respectively). While two monomers are bridged by a single semi-rigid bond for P and by a flexible long linker for Pc, the two monomeric units of Ru(phen)_2_ip^2+^ in Pi are linked by two single bonds via a benzene ring (orange), providing additional rotational degrees of freedom. Previous bulk measurements reported by Chao *et al*. showed that Pi elongates DNA^16^, but linear dichroism (LD) experiments by Andersson *et al*. found that the DNA-Pi complex does not preserve the free-DNA orientation, in contrast to conventional intercalators^15^. In addition, a ligand/base-pair ratio of 1:10 resulted in complete DNA condensation, limiting the ability of bulk experiments to characterize the Pi-DNA intercalation mechanism^15^.

Strong DNA condensation by cationic ligands with high affinity to DNA poses a great challenge in bulk experiments^15^,^17^. However, in single molecule experiments the ends of the DNA molecule are pulled apart, preventing the molecule from dropping out of solution when condensed, and preventing condensation at high pulling forces^18^. In addition, in single molecule experiments a much smaller concentration of ligands is required to observe significant binding, which also greatly diminishes DNA condensation^19^. We used dual-beam optical tweezers to conduct single-molecule force spectroscopy experiments using a force clamp, allowing us to fully characterize the affinity, kinetics, and the governing structural dynamics of this unique intercalating system. We found that this ligand possesses the highest DNA binding affinity measured with this method, combined with one of the slowest dissociation rates from the final intercalative state (Fig. 1A).

## Results

### Rapid intercalation followed by slow conversion to a fully intercalated state

We examined the kinetics of Pi interacting with single λ-DNA molecules as a function of constant applied forces of 10 to 50 pN and ligand concentrations of 0.15 to 40 nM. Figure 2A shows force clamp measurements in which DNA-ligand intercalation is monitored over tens of minutes, starting from the free DNA extension until the DNA-ligand complex reaches equilibrium. The DNA elongation measurements illustrate two distinct phases during association; rapid intercalation that is analogous to conventional intercalation, followed by very slow intercalation that approaches equilibrium with a rate that is comparable to that observed for other threading intercalators^5^,^11^,^23^. We then measured Pi dissociation from DNA after rinsing the binding ligands from the surrounding solution (Fig. 2B), and observed that the DNA-ligand complex extension decreases to the DNA-only extension over a timescale longer than the association process. Interestingly, the dissociation measurements fit well to a single rate that is comparable to the dissociation rate estimated for the threading mono-intercalator P^5^,^23^.

**Figure 1:**
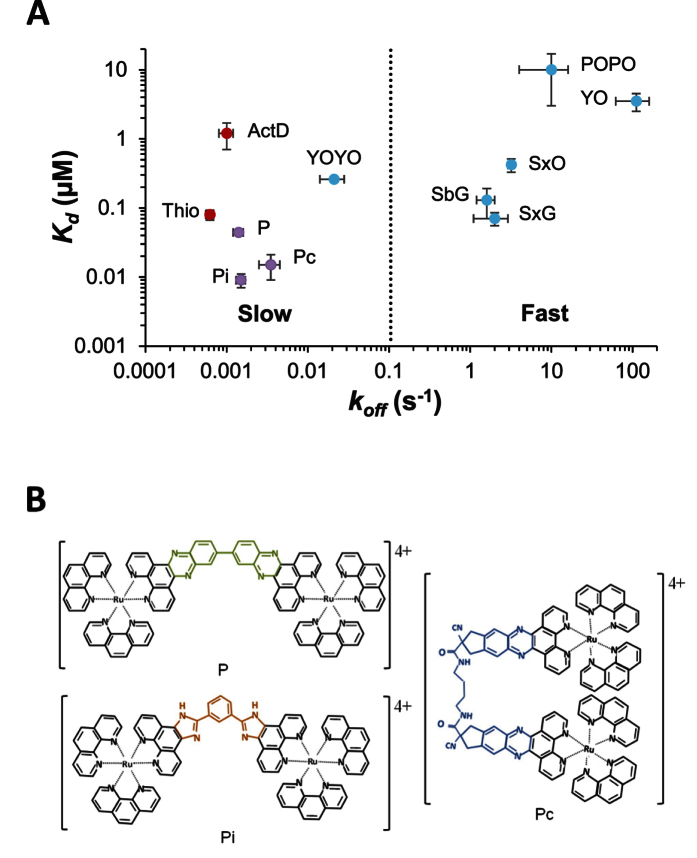
(**A**) Plot of the equilibrium dissociation constant *K*_d_ and the dissociation rate from the final equilibrium state *k*_off_. Cyanine dye (cyan symbols) values were reported by Biebricher *et al*.^12^, except for the *K*_d_ of YOYO, which is from Murade *et al*.^14^. Polypeptides (red symbols) are from Camunas-Soler *et al*.^13^ and Paramanathan *et al*.^7^ for Thiocoraline (Thio) and actinomycin D (ActD), respectively, threading intercalators (purple data) are from previous reports by Almaqwashi *et al*.^5^, Bahira *et al*.^11^ and this work for P, Pc, and Pi, respectively. The error bars are from the reported values of previous studies, except the values for Pi which are determined in this study. (**B**) Binuclear ruthenium complexes Δ,Δ-[μ-bidppz(phen)^4^Ru_2_]^4+^ (P), Δ,Δ-[μ-C4(cpdppz)_2_(phen)_4_Ru_2_]^4+^ (Pc), and the ligand investigated here Δ,Δ-[μ-bipb(phen)_4_Ru_2_]^4+^ (Pi). Differences in intercalating moieties are highlighted in green, blue, and orange, respectively.

**Figure 2:**
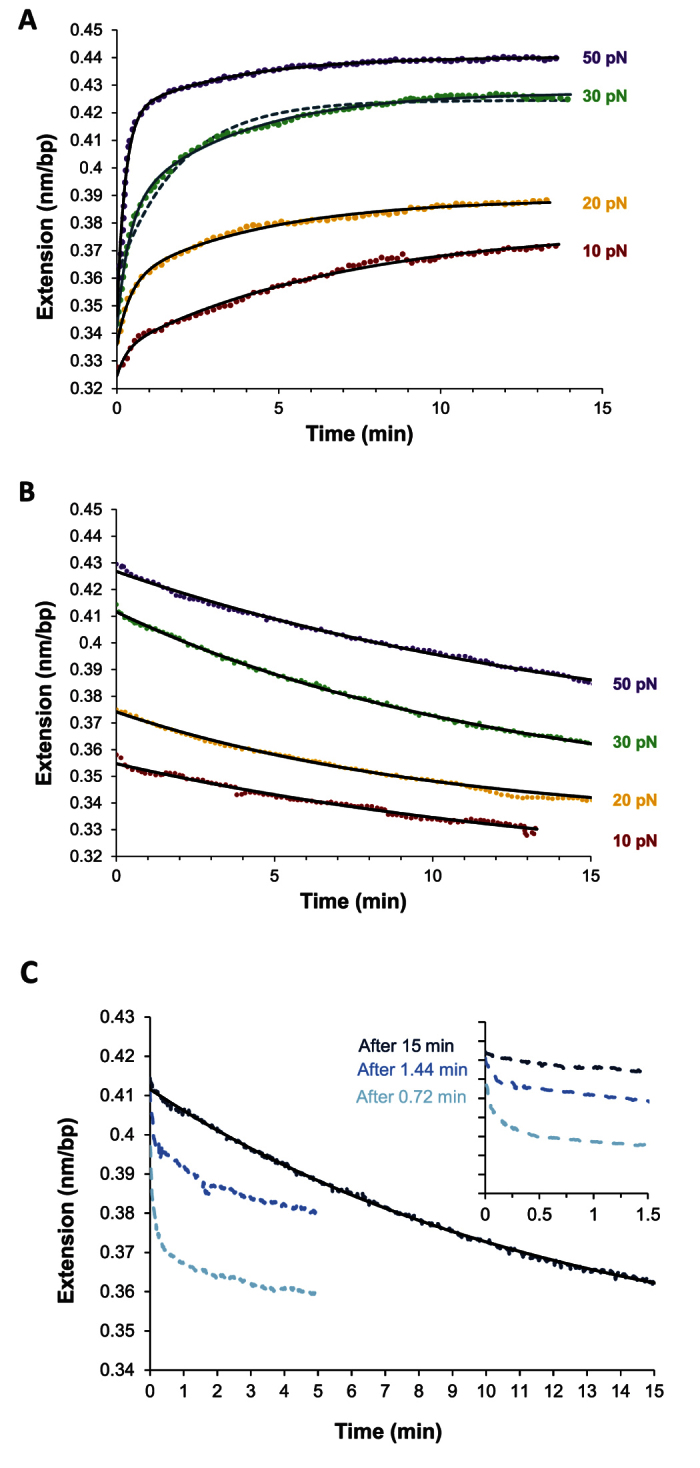
(**A**) The time-dependent equilibrium extensions of the DNA-Pi complex at Pi concentration of 5 nM and constant forces 10 pN to 50 pN as color coded, where dots are data, and solid lines are fits to a double exponential time dependence (see Methods). For comparison, a fit to a single exponential time dependence is presented as a dashed line for 30 pN. (**B**) The time-dependent extensions of DNA-Pi complex dissociation fitted with a single exponential rate, giving a direct measurement of the off rate from the final state at each applied force as color coded. (**C**) Demonstration of the validity of a three-state model by stopping the association after 0.72 min, 1.44 min and 15 min of ligand flow incubation. Rinsing the ligand out of solution after a short flow time reveals a fast dissociation time of less than 0.5 min. The larger fraction of the ligand that dissociates on a less than one minute time scale suggests that the conversion of the bound ligand from its fast intermediate intercalated state (I^ǂ^) to its slow final intercalated state (I) does not occur during this short ligand flow incubation time.

In order to test our hypothesis of rapid formation of an intermediate state followed by slow formation of a final intercalated state, we probed the occupancy of the intermediate state by stopping the intercalation process at intermediate times and washing off the ligand (Fig. 2C). We observe a growing fraction of fast dissociating ligand when the ligand flow incubation time becomes shorter than the time needed for Pi to convert from its intermedate intercalated state to its final intercalated state, which occurs on a ~100 s time scale. The fast dissociation time from the intermediate intercalated Pi state is less than ~10 s, which is in the range of dissociation rates for conventional DNA intercalators (Fig. 1A). After an incubation time of tens of minutes, the amplitude of the fast dissociation fraction vanishes, indicating that all ligands are now in their final intercalated state. This is consistent with Pi conversion from a rapidly forming conventional intercalated state to a slowly forming unconventional, final threaded, intercalated state. These results in turn show that the intermediate state is in pre-equilibrium, an approximation employed below in analyzing the kinetics of this complex system^5^,^11^,^19^. After the conversion is completed (Fig. 2C, dark blue measurement), the slow Pi dissociation reflects the unthreading process, involving the energetically costly melting of several DNA base pairs.

### Two-step kinetics analysis reveals fundamental DNA intercalation rates and affinities

**Figure 3:**
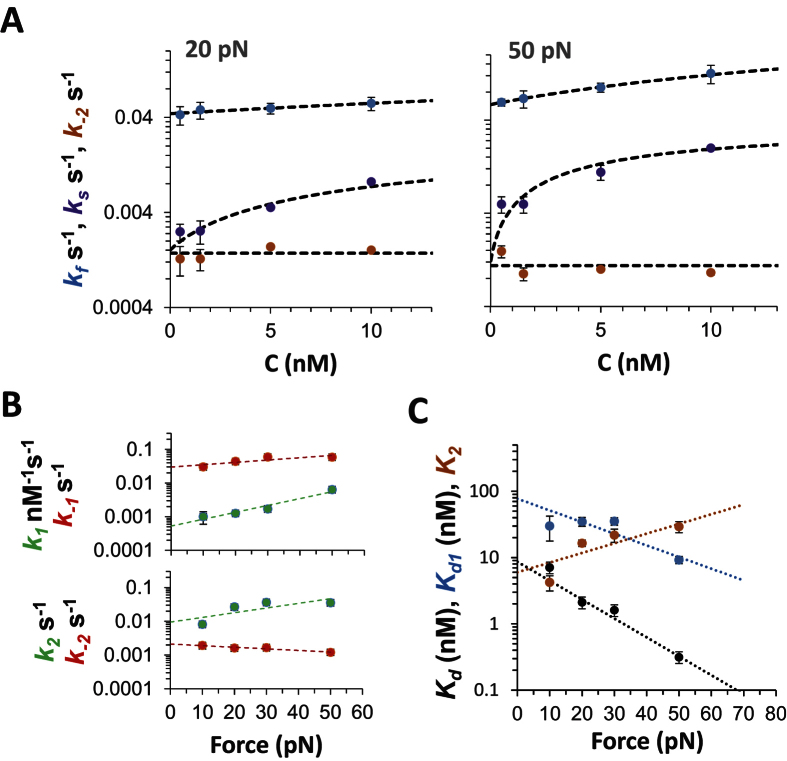
Kinetics of Pi binding to DNA. (**A**) Examples of measured *k*_*f*_, *k*_*s*_. and *k*_−*2*_ rates (as color coded) at constant forces of 20 pN and 50 pN as a function of Pi concentration fitted to Eq. 3, as described in the Methods section. As expected the measured *k*_−*2*_ values show no Pi concentration dependence. The error bars are standard error determined directly from experimental repetition of the measurements. (**B**) Values of elementary rates *k*_*1*_, *k*_−*1*_. *k*_*2*_and *k*_−*2*_ (as color coded), obtained from the measured *k*_*f*_, *k*_*s*_. and *k*_*-2*_ rates, are fitted to the exponential force dependence, as described by Eq. 5. The elementary rates extrapolated to zero-force are summarized in Table 1. (**C**) Force-dependent binding constants *K*_*d1*_, *K*_*2*_ and *K*_*d*_ (as color coded) determined from the elementary rates fitted to an exponential force dependence. The fitted zero-force values of the equilibrium dissociation constants for the whole intercalation process, and for each of its steps, as well as associated equilibrium length changes, are also collected in Table 1. The error bars in B and C correspond to a confidence level of 68% in constant chi-squared boundaries.

The traditional single transition model of a mono-intercalating system is not found to reflect the observed kinetics, as two rates are required to adequately fit the data (see Fig. 2A, dash line fit). We therefore used a two-step kinetics analysis^11^,^20^,^21^ (outlined in Methods) to analyze the fast (*k*_*f*_) and slow (*k*_*s*_) association rates obtained from the double exponential fits of DNA-Pi complex time-dependent extension measured at constant force. In this model, an initial fast, bimolecular binding event is followed by a slower, unimolecular binding event, as demonstrated above. Fits to this model reveal the fundamental reaction rates for both steps of this process, *k*_*1*_*c*, *k*_−*1*_, *k*_*2*_ and *k*_−*2*_, where *k*_*1*_*c* and *k*_*−1*_ represent association and dissociation to and from the intermediate intercalated state (I^‡^), while *k*_*2*_ and *k*_−*2*_ represent association and dissociation to and from the final (I) intercalated threaded state (see Methods, Eq. 2). Figure 3A shows fits to the concentration dependence for the measured *k*_*f*_ (C) and *k*_*s*_ (C) at two constant forces, while dissociation experiments that directly determine *k*_−*2*_ show no concentration dependence, as expected for a unimolecular dissociation rate. The elementary rates *k*_*1*_, *k*_−*1*_, *k*_*2*_ and *k*_−*2*_ are then fit to an exponential force dependence (Methods Eq. 5), as shown in Fig. 3B, revealing the zero-force rates and their associated DNA extension lengths (Table 1). The elementary rates, in turn, allow determination of the equilibrium constants *K*_*d1*_, *K*_*2*_ and *K*_*d*_ for the first and the second intercalation steps, as well as for the complete process of DNA-Pi threading. The force-dependent equilibrium constants are then fit to an exponential force dependence as shown in Fig. 3C, yielding the zero-force equilibrium constants and kinetic rates as well as their related DNA deformation lengths. These results, which validate the assumption that the intermediate state achieves rapid equilibrium before binding is complete, are summarized in Table 1.

### Equilibrium parameters from DNA-Pi complex elongation confirm kinetics results

**Figure 4:**
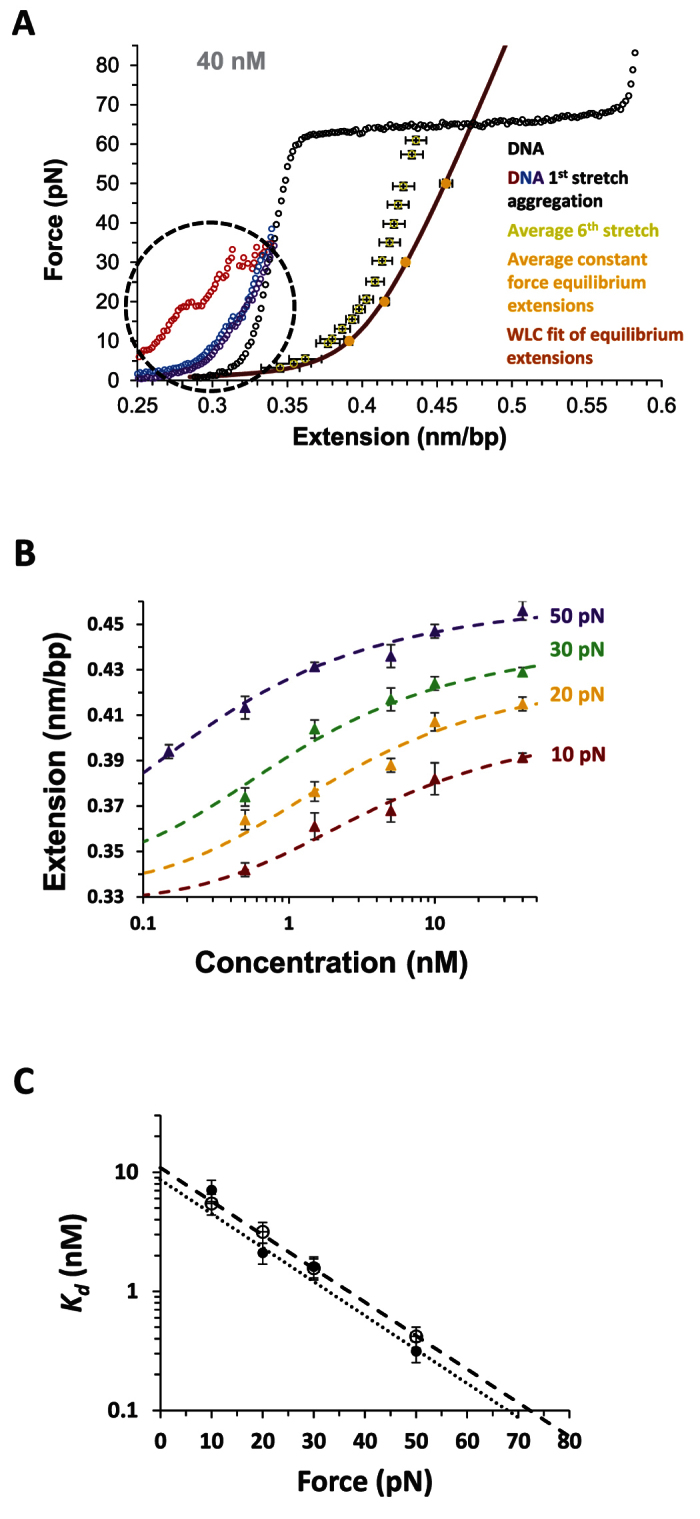
(**A**) As color coded: WLC fit of the saturated DNA-Pi complex extensions. The Pi-DNA complex aggregates at low forces (and low extensions), in agreement with the bulk solution observations, as observed during the 1^st^ stretch for each of the three different DNA molecules (shown in different colors in the dashed circle). The second stretches (not shown) show much less aggregation, and successive stretches show only DNA elongation due to the fast phase intercalation, since each individual stretch takes only ~100 s. The average of the 6^th^ stretch for different molecules shows that the Pi-DNA complex is still far from its equilibrium extension, when compared to the equilibrium F(x) dependencies obtained from the force clamp measurements at each Pi concentration. Fitting the equilibrium extensions at saturated concentration to the WLC model (Methods, Eq. 7) yields the saturated Pi-DNA complex persistence length *L*_p_ = 10 ± 2 nm and elastic modulus *S* = 414 ± 40 pN. (**B**) Measured equilibrium DNA-Pi complex extensions for 10–50 pN forces and Pi concentrations of 0.15–40 nM, along with their fits to the McGhee-von Hippel binding isotherm, yielding dissociation constants *K*_d_(*F*) ranging from 0.42 nM at 50 pN to 5.47 nM at 10 pN, with the constant binding site size *n* = 2.8, optimizing the global fit at all forces. The value *n* = 2.8 ± 0.3 bp is in good agreement with *n* = 3.3 ± 0.4 obtained from the saturated binding, 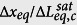. The error bars in A and B are standard error determined directly from the experimental repetition of the measurements. (**C**) *K*_*d*_(F) from the equilibrium measurements (dashed fit line and open circle data) agrees closely with the *K*_*d*_(F) values determined from the measured reaction rates (dotted fit line and solid circle data), both fitted to the exponential force dependence (Eq. 6). The error bars correspond to a confidence level of 68% in constant chi-squared boundaries.

The force-extension curve obtained for the saturated DNA-Pi complex, 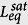, is fit to the WLC model^20^,^21^,^22^ (Fig. 4A), as described in Methods. The obtained equilibrium elastic properties of the saturated DNA-Pi complex, including its contour length, persistence length, and elastic modulus, are comparable to values of these parameters previously measured for threading intercalators^23^,^24^. Figure 4A also shows the effects of aggregation on the DNA stretching curves, which can only be observed at high concentrations when holding DNA at very low initial extensions, comparable to bulk experimental conditions, which also observed aggregation^15^. During the first DNA stretch in the presence of ligand in solution on the time scale of ~100 s, the DNA elongation due to initial fast intercalation is observed, but the final state is not reached even after several consecutive stretch and release cycles. The equilibrium extensions of the DNA-Pi complex can only be obtained after Pi-DNA incubation for more than 10 min at each ligand concentration and applied DNA stretching force. These measurements determine the *L*_*eq*_(*C*) values presented in Fig. 4B for the four different force values, which give the DNA-Pi titration curves that can further be fit to the McGhee-von Hippel (M-H) model^5^,^25^,^26^,^27^,^28^, as described in Methods, to obtain the equilibrium constant *K*_*d*_ at each applied force, plotted in Fig. 4C. The fit of the *K*_*d*_(*F*) dependence as obtained in this equilibrium analysis confirms the *K*_*d*_(*F*) values determined from the fitted elementary reaction rates from our kinetics measurements. The zero-force equilibrium dissociation constant *K*_*d*_(0) = 11 ± 2 nM obtained from this equilibrium analysis is consistent with its value determined from kinetics measurements. In addition, we determine the equilibrium DNA elongation upon complete Pi threading to be Δ*x*_eq_ = 0.27 ± 0.03 nm.

## Discussion

The DNA intercalation affinity found here for the two-step threading of Pi is ~5-fold higher than that of its closely related parent molecule P, which exhibits one-step intercalation in single molecule studies^5^,^7^,^12^. The quantified dynamic DNA deformations show that two-step intercalation also exhibits a molecular lock mechanism, in which equilibrium DNA deformation in the final state is less than the dynamic DNA deformation that is required for the full DNA-ligand assembly process. Thus, while the overall DNA elongation in the forward transition is *x*_*on*_ = *x*_+1_ + *x*_+2_ = 0.35 nm, the DNA- Pi complex is relaxed to Δ*x*_eq_ = 0.27 nm in the final equilibrium state. These findings confirm a previous prediction by Bahira *et al*.^11^ that combining two-step intercalation^11^,^13^,^29^ with a molecular lock mechanism^5^,^7^ would result in higher DNA intercalation affinity than that observed for each of these properties alone. The first intercalative step of the binuclear complex Pi has an equilibrium constant of ~80 nM, which is close to the ~100 nM equilibrium constant reported in bulk for conventional DNA intercalation by an analogous mononuclear version of Pi (which has only one of the bulky threading units, enabling the exposed benzene ring to intercalate conventionally)^30^. This shows that the additional requirements of DNA deformation and ligand threading for the binding of Pi strongly enhance its affinity by an order of magnitude relative to intercalation by the benzene ring alone.

**Figure 5:**
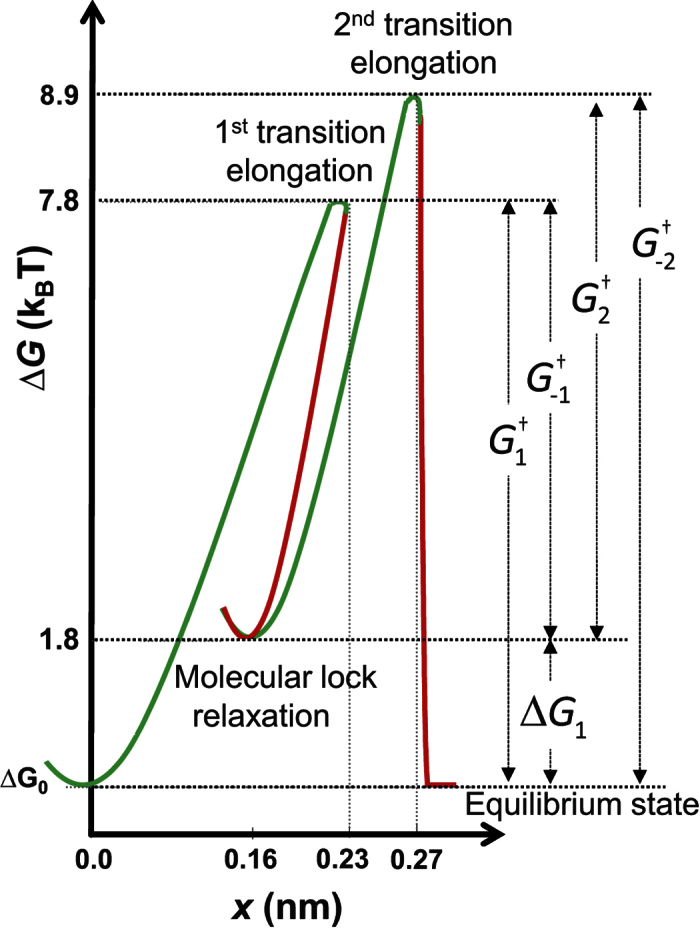
Illustration of the two-step lock mechanism leading to the final intercalated state of the DNA-Pi complex. The non-intercalated state (NI) and final intercalated state (I) have the same free energy reference value ∆G_0_ at ligand concentration equal to the equilibrium dissociation constant *K*_d_. The intermediate intercalated state sits at a free energy higher than ∆*G*_0_ by Δ*G*_1_ = *k*_*B*_*T* · ln(*K*_2_(0)) = 1.8*k*_*B*_*T*. Assuming that the attempt rate for the initial bimolecular association process is approximately *k*_*diff*_ = 10^9^ M^−1^s^−1^, this leads to a first transition free energy barrier of 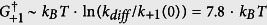. This information allows us to determine the reverse barrier for the first transition 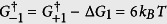. The second barrier height then becomes 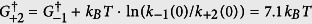. These calculations uniquely determine all of the free energy landscape parameters shown in the figure.

**Figure 6:**
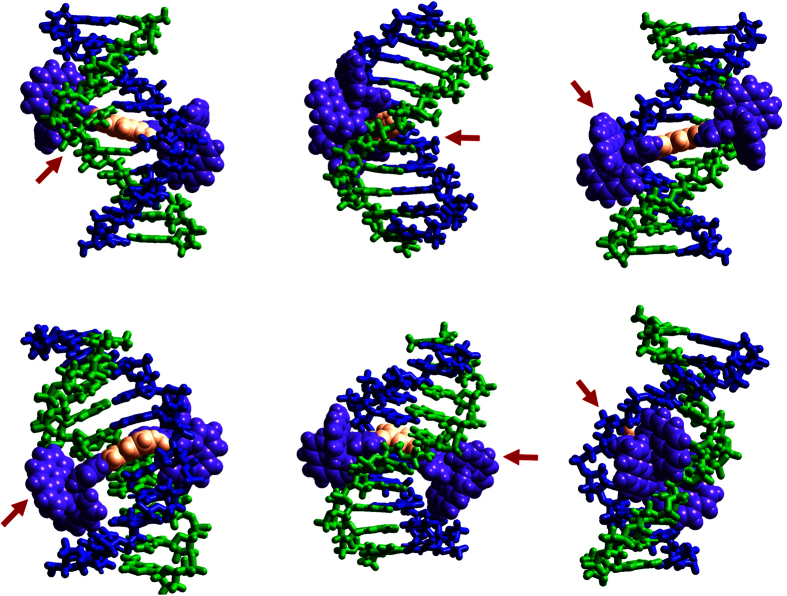
Cartoons showing possible binding geometries of Pi to (TCGGTACCGA)_2_. Left: major groove view; middle: side view; right: minor groove view. First row: intercalation from the minor groove, Second row: threading intercalation. Note that the bridging ligand is non-planar due to rotation about the single bonds. The benzene ring (orange) is stacked between A and T on the same strand. The bridging ligand is planar, and symmetrically disposed in the minor groove. Also shown here is the benzene ring, the only properly intercalating moiety, stacked between A from opposite strands. The red arrows point to structural differences between the intial intercalation from the minor groove and the final threading intercalation state. The pictures were obtained by several steps of manual docking and subsequent energy minimization in vacuo using the Amber force field in the HyperChem software package (HyperCube Inc.). The charge of the complex was set to zero to mimic the electrostatic screening of the buffer.

The results reveal a model for DNA-Pi assembly, illustrated in Fig. 5 based on the free energy landscape of this unique double-transition mono-intercalating system. In this proposed intercalation mechanism, the rapidly forming intermediate intercalative state represents conventional intercalation by the benzene ring, leading to unwinding of the double helix, which makes the subsequent threading transition an order of magnitude energetically more favorable than the threading of the same dumbbell moiety in the threading intercalator P. Following the threading transition, the DNA-Pi complex reaches an equilibrium state in which DNA intercalation by the benzene ring is optimized. Figure 6 approximately illustrates the Pi intermediate (top row) and final intercalating states (bottom row), where only the bridging benzene ring is the properly intercalating moiety. Note that the rotational flexibility of Pi may facilitate intercalative minor groove binding, the transition from the intermediate state to the final state, and the accommodation of both ends of the dumbbell in the minor and major grooves. This outlined intercalation mechanism indicates that the intercalating moiety is partially inserted between two initial base pairs, then the threading transition occurs between adjacent base pairs, resulting in stacking with the adjacent bases of the larger aromatic ring area of the ligand, thereby leading to the higher affinity state. The slowness of the second step is associated with threading of the bulky Ru(phen)_2+_ moiety through the DNA base pair, requiring major duplex disruption. However, threading of this pre-intercalated DNA duplex is ~10-fold faster than the threading intercalation of the P ligand (compare k_2_ for Pi and k_1_∙K_d_ = k_−1_ for P), and overall ~5-fold more driven.

These novel findings for Pi not only overcome the limitation of bulk measurements in resolving the binding mode of the DNA-Δ,Δ-Pi interaction, but also present a convincing illustration of the utilization of single-molecule studies to provide important insights for the rational design of DNA-targeting ligands. The specific mechanism dissected here represents the first measurement of a two-step intercalator combined with a molecular lock mechanism. This mechanism results in the highest affinity measured for such an unconventional intercalator, as well as one of the slowest binding mechanisms observed. Using this information, DNA-ligand structural dynamics may be optimized for effective antitumor treatment in the presence of other non-intercalating DNA-targeting agents.

## Methods

### Experimental measurements

All experiments were conducted using dual-beam optical tweezers (laser wavelength 830 nm). A single bacteriophage λ-DNA, labeled on opposite strands with biotin, was attached between two streptavidin-coated polystyrene beads (~5.6 μm); one bead is held in the optical trap and the other bead is held by a glass micropipette. A piezoelectric positioner displaces the micropipette (±10 nm) to maintain a fixed stretching force (±1 pN) on the DNA molecule. After the attachment between the two beads, the DNA-only stretching curve is obtained at a pulling rate of ~200 nm/s. Then, the DNA is stretched rapidly (~2 s) to reach the assigned constant force and the elongation from the DNA-only equilibrium extension due to the threading intercalation by Pi is traced. After the DNA-Pi complex reaches equilibrium elongation, the ligand is rinsed out by flowing ligand-free buffer solution. As the ligand dissociates, the DNA-Pi complex elongation is traced back to the free DNA extension. The force feedback reacts to any sudden force change as fast as 50 ms by displacing the micropipette to maintain the assigned force. The experiments were carried out in a 100 μl flow cell chamber volume and a constant ligand flow rate of ~2 μl/s. Constant force measurements were obtained on at least three DNA molecules for each averaged data point. All measurements were obtained at 21 °C and under buffer conditions of 10 mM Tris, 100 mM NaCl and pH 8. Pi was synthesized and purified as described elsewhere^15^.

### Kinetics rate analysis

The time-dependent DNA elongations are fit to a double-exponential dependence on fast and slow rates.


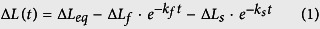


The observed fast and slow rates are related to the elementary rates of two-step DNA intercalation^11^,^31^,^32^.


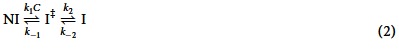


For the conditions (*k*_*1*_ · *C* + *k*_−*1*_ ≫ *k*_*2*_ + *k*_−*2*_), in which the non-intercalative state (NI) and the intermediate intercalative state (I^‡^) states rapidly equilibrate before the second transition to the final intercalative state (I), we can then fit the measured fast and slow rates in terms of the elementary rates.


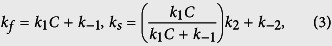


From the elementary rates we determine the equilibrium constants of the first and second transition as well as the final equilibrium state, respectively:


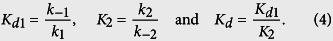


The zero-force rates, zero-force equilibrium constants, and their DNA deformation lengths are obtained from chi squared minimized fitting to an exponential dependence on force^3^,^4^.


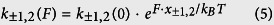



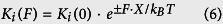


Here *K*_i_ is *K*_d1_, *K*_2_ or *K*_d_ and *X* is *x*_1_, *x*_2_ or ∆*x*_eq_, respectively. It is important to note that the force dependence resulting from the structural elongation is a property only of the elementary rates. It is these elementary rates that determine the free energy landscape, rather than the overall measured fast and slow intercalative rates.

### Equilibrium extension analysis

The saturated DNA-ligand extensions from force clamp measurements are fit to the Worm-like chain model of polymer elasticity^25^.


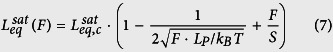


The fractional lengthening directly corresponds to the fractional ligand binding, 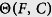, for a particular Pi concentration at a fixed force, and is given by the ratio of the lengthening observed due to the binding of the intercalator ∆*L*_*eq*_(*F,* C) to the complex lengthening observed at saturated ligand binding, 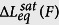:


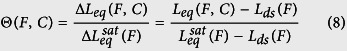


We fit our measured fraction of ligand bound to McGhee-von Hippel (M-H) binding isotherm^5^,^25^,^26^,^27^,^28^


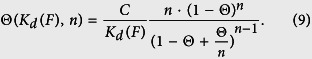


Eqs (8) and (9) can be substituted into eq. (7) to calculate *L*_*eq*_ as a function of concentration at each constant force. Matching these calculated and measured *L*_*eq*_(*F*, *C*) values allows us to determine the equilibrium dissociation constant *K*_d_(*F*) and the intercalative occluded site size *n*.


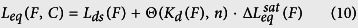


The force-dependent *K*_d_(*F*) values are then fit to the exponential force dependence as given by Eq. 6.

## Additional Information

**How to cite this article**: Almaqwashi, A. A. *et al*. DNA intercalation optimized by two-step molecular lock mechanism. *Sci. Rep.* **6**, 37993; doi: 10.1038/srep37993 (2016).

**Publisher's note:** Springer Nature remains neutral with regard to jurisdictional claims in published maps and institutional affiliations.

## Figures and Tables

**Table 1:** Zero-force equilibrium and kinetics parameters for Pi-DNA intercalation, obtained from the kinetics measurements.

Complex	Equilibrium parameters from fundamental rates							
	*K*_*d*_ (nM)	Δ*x*_eq_ (nm)	*K*_*d1*_ (nM)	*x*_1_ (nm)	*K*_*2*_ (-)	*x*_2_ (nm)		
P	44 ± 2	0.19 ± 0.01	—	—	—	—		
Pc	15 ± 6	0.44 ± 0.04	35 ± 9	0.24 ± 0.02	1.8 ± 0.6	0.22 ± 0.03		
Pi	9 ± 2	0.27 ± 0.03	77 ± 10	0.17 ± 0.02	6 ± 1	0.14 ± 0.02		
	Kinetics parameters							
	***k***_***+*****1**_**(0) (M**^**−1**^**s**^**−1**^**) × 10**^**5**^	***x***_***+*****1**_**(nm)**	***k***_**−*****1***_**(0) (s**^**−1**^**) × 10**^**−3**^	***x***_**−1**_ **(nm)**	***k***_***+*****2**_**(0) (s**^**−1**^**) × 10**^**−3**^	***x***_***+*****2**_ **(nm)**	***k***_**−*****2***_**(0) (s**^**−1**^**) × 10**^**−3**^	***x***_***−*****2**_ **(nm)**
P	0.3 ± 0.03	0.33 ± 0.02	1.4 ± 0.1	0.14 ± 0.01	—	—	—	—
Pc	18 ± 0.4	0.19 ± 0.02	68 ± 4	−0.06 ± 0.01	6 ± 1	0.08 ± 0.01	3.6 ± 1.0	−0.15 ± 0.03
Pi	4 ± 0.03	0.23 ± 0.02	28 ± 2	0.07 ± 0.01	9 ± 1	0.12 ± 0.01	1.5 ± 0.2	0.0 ± 0.01

Previously reported values and error bars of P and Pc are from Almaqwashi *et al*.^5^,^23^ and Bahira *et al*.^11^, respectively. For Pi, The error bars correspond to a confidence level of 68% in constant chi-squared boundaries.
